# Effect of Elevated CO_2_ Concentration on the Disease Severity of Compatible and Incompatible Interactions of *Brassica napus*–*Leptosphaeria maculans* Pathosystem

**DOI:** 10.3390/plants8110484

**Published:** 2019-11-08

**Authors:** Zhongwei Zou, Fei Liu, Changqin Chen, W. G. Dilantha Fernando

**Affiliations:** 1 Department of Plant Science, University of Manitoba, 66 Dafoe Road, Winnipeg, MB R3T 2N2, Canada; Zhongwei.Zou@umanitoba.ca (Z.Z.); Fei.Liu@umanitoba.ca (F.L.); chenchangqin@jlau.cn (C.C.); 2 College of Plant Protection, Jilin Agricultural University, Changchun, Jilin 130118, China

**Keywords:** *Leptosphaeria maculans*, elevated CO_2_, *Brassica napus*, climate change, plant disease

## Abstract

Global warming by increased atmospheric CO_2_ concentration has been widely accepted. Yet, there has not been any consistent conclusion on the doubled CO_2_ concentration that in the future will affect plant disease incidence and severity. Blackleg disease, mainly caused by *Leptosphaeria maculans*, is a major disease on canola production globally. *Brassica napus* and *L. maculans* have a gene-for-gene interaction, which causes an incompatible reaction between canola plants carrying resistance genes and *L. maculans* isolates carrying corresponding avirulence genes. In this study, *B. napus* varieties and lines inoculated with different *Leptosphaeria* isolates were subjected to simulated growth conditions, namely, growth chambers with normal environments and with controlled CO_2_ concentrations of 400, 600, and 800 ppm. The results indicated that the elevated CO_2_ concentrations have no noticeable effect on the inferred phenotypes of the canola–blackleg interactions. However, the disease severity decreased in most of the *B. napus*–*L. maculans* interactions at extremely high CO_2_ concentration (800 ppm). The varied pathogenicity changes of the *B. napus–L. maculans* pathosystem under elevated CO_2_ concentrations at 400 or 600 ppm may be due to the genetic background or physiological differences in plants and pathogenicity differences in *L. maculans* isolates having different *Avr* gene profiles. The mechanisms by which elevated CO_2_ concentrations affect the *B. napus–L. maculans* pathosystem will help us understand how climate change will impact crops and diseases.

## 1. Introduction

Climate change is an important dynamic that will affect food production globally. The atmosphere’s elevated concentration of CO_2_, one of the most important climate change influences, is due to anthropogenic emissions [1]. The concentration of CO_2_ has been increasing and is predicted to double at the end of this century by the Intergovernmental Panel on Climate Change (IPCC) [2]. Levels will climb from the current level of 380 µmol/mol to 730–1020 µmol/mol [3]. The effects of rising CO_2_ concentrations on crop growth and physiological processes have been extensively studied [4,5,6,7]. For instance, rising CO_2_ can enhance the physiological performance of *Brassica napus* seedlings under optimal water supply [8]. Li et al. (2017) reported that increased CO_2_ could alter green tea quality by the stimulation of primary and secondary metabolism [9]. They found that the higher concentration of CO_2_ could enhance photosynthesis, C:N ratio, Rubisco carboxylation activity, water-use efficiency, and final yield or quality [6,8,9,10,11].

Plant disease symptoms in pathosystems are influenced by, or require, three components—a susceptible host, an aggressive or virulent pathogen, and a conducive environment for the pathogen to cause disease [12,13]. Thus, the variations in environmental conditions, including CO_2_ concentration change, may potentially affect plant disease susceptibility and severity. A couple of studies have investigated the elevated CO_2_ concentration effects on plant–pathogen interactions. The exposure of tomato plants to elevated CO_2_ led to a lower disease incidence and severity caused by tomato (*Solanum lycopersicum*) mosaic virus (TMV) and *Pseudomonas syringae*. However, the plant’s susceptibility to necrotrophic *Botrytis cinerea* was increased [14]. Ferrocino et al. (2013) found that increased CO_2_ concentration had no detectable impact on the Fusarium wilt of lettuce (*Lactuca sativa*) or the abundance of *Fusarium* spp. [15]. The leaf spot disease incidence and severity always increased in redbud (*Cercis canadensis*) and sweetgum trees (*Liquidambar styraciflua*) under increased CO_2_ concentration in a five–year survey [16]. Elevated CO_2_ reduced the disease incidence and severity significantly in the red maple fungal pathogen *Phyllosticta minima* [13]. The species diversity of foliar fungal plant disease decreased when the CO_2_ concentration increased [17]. The disease incidence and severity of rocket plants (*Eruca sativa*) caused by *Fusarium oxysporum* increased under elevated CO_2_ conditions with controlled temperatures [18]. Considering the above, it is difficult to find a consistent pattern to the effects of elevated CO_2_ concentration on disease incidence and severity in plants.

Canola (*Brassica napus*) is one of the most important cash crops in Canada, reaching a production of 20.3 MMT in 2018 (Canola Council of Canada). Blackleg, caused by the fungal pathogen *Leptosphaeria maculans*, is one of the most important and devastating diseases in canola worldwide, which causes significant yield loss [19]. The incidence and severity of blackleg disease in canola vary with the geographic distribution, varieties, crop rotation, and climate conditions. This disease has been controlled historically by crop rotation and the utilization of canola varieties with major gene resistances [20,21]. A gene–for–gene interaction has been reported between *B. napus* with a certain *R* gene and its corresponding avirulence gene (*Avr*) in the *L. maculans* isolate [22,23]. This pathosystem brings incompatible and compatible interactions in the canola plant, with and without a certain *R* gene, respectively, that can defend against the *L. maculans* pathogen with the corresponding *Avr* gene. Currently, a total of 16 *Avr* genes have been identified in *L. maculans* [24,25,26,27,28]. Corresponding to the *Avr* genes in *L. maculans*, various major resistance genes in *Brassica* spp. have been identified [29,30,31,32,33]. Apart from the qualitative resistance (major gene resistance), the effect of quantitative resistance against an isolate of *L. maculans* in selected Canadian canola cultivars under increased temperature was investigated [34]. Very few studies have reported on the effects of environmental changes, especially the CO_2_ concentration, on the interactions of the *B. napus–L. maculans* pathosystem. In this study, we conducted experiments in controlled-environment rooms with three different CO_2_ concentrations of 400, 600, and 800 ppm. The aims were to understand the effects of elevated CO_2_ on (1) the compatible and incompatible interactions between *B. napus* and *L. maculans* isolates; (2) the disease severities of the susceptible and resistant canola varieties; and (3) the pathogenicity of different isolates carrying different *Avr* gene races.

## 2. Results

### 2.1. Inferred Phenotyping

The mean rating scores and their inferred disease resistance for the seedlings of *B. napus* varieties and lines had different variations based on the inoculum and CO_2_ concentrations (Table 1). *L. maculans* isolate D5, which carries *AvrLm1-2-4-7-S-LepR1-LepR2*, showed susceptible (S) and intermediate resistance (IR) on *B. napus* line 1065 under CO_2_ concentrations of 400 and 800 ppm, respectively, whereas it showed a resistance (R) reaction under a normal environment (NE) and a CO_2_ concentration of 600 ppm. The inferred plant phenotypes of *L. maculans* isolate D10 (*AvrLm5-6-7-LepR1*) inoculated onto *B. napus* varieties and lines 1135 and 01-23-2-1 carrying *LepR2* and *Rlm7* genes, respectively, were changed from susceptible into intermediate resistance at the highest CO_2_ concentration (800 ppm). The DM118 isolate showed a high disease rating score of 6.00 (IR) at the 600 ppm concentration of CO_2_ when inoculated onto the *B. napus* 1065 line. The disease rating score was decreased to 5.25 under 800 ppm CO_2_ when the DS103 isolate was inoculated onto *B. napus* line 1065. A CRISPR mutant isolate umavr7 produced from DS103 showed intermediate resistance (5.25) in *B. napus* variety or line 1065 under 400 ppm, reduced from a susceptible reaction (8.33, NE; 6.67, 600 ppm; 8.00, 800 ppm). In addition, it was also reduced when CO_2_ concentrations increased from 400 to 800 ppm (Table 1). Usually, the *L. biglobosa* isolate is considered as non–aggressive or less virulent to *B. napus*. In this study, we found that *L. biglobosa* showed intermediate resistance on *B. napus* line 1135 under a normal environment and 600 ppm CO_2_, and under a normal environment in both *B. napus* varieties and lines 02-22-2-1 and Goé Land (Table 1).

### 2.2. Pathogenicity Evaluation of the Susceptible B. napus Variety Westar

**Figure 1:**
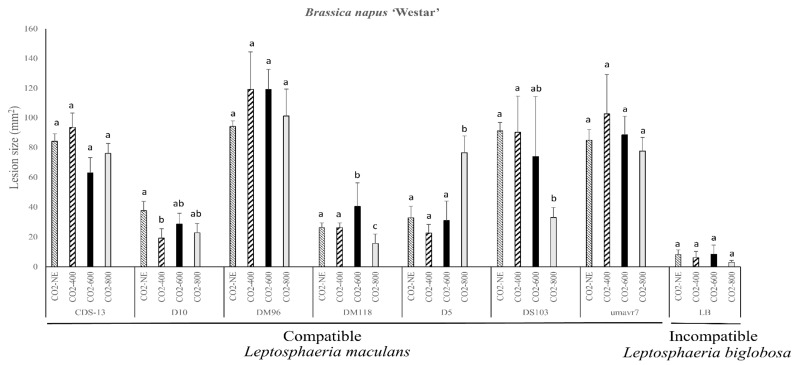
Pathogenicity assessment of seven *Leptosphaeria maculans* isolates inoculated onto *Brassica napus* variety Westar under different CO_2_ concentrations (NE, normal environment; CO_2_-400, 400 ppm; CO_2_-600, 600 ppm; CO_2_-800, 800 ppm). A less virulent or non-aggressive isolate *Leptosphaeria biglobosa* was included as a control. The lesion sizes were quantified at 14 days post-inoculation (dpi) for each treatment using Assess 2.0 (American Phytopathological Society, St. Paul, MN, USA). Statistical analysis was performed using SAS version 9.4 (SAS Institute, Inc., Cary, NC, USA) for analysis of variance (ANOVA). Each bar indicates the mean and standard deviation of the lesion size for a particular isolate at different CO_2_ concentrations. Different letters over the bars represent the significant differences (*p* < 0.05) in the lesion sizes caused by a specific isolate under four different CO_2_ environments.

The *L. biglobosa* isolate, which is avirulent to the canola plants, did not show any significant lesion size change under different CO_2_ concentrations on *B. napus* variety Westar. The lesion size decreased slightly when the CO_2_ concentration was increased to 800 ppm (Figure 1). All the other seven involved *L. maculans* isolates showed virulence and clear disease symptoms on *B. napus* variety Westar. The cotyledon lesion size caused by *L. maculans* isolate D10 was significantly decreased under 400 ppm of CO_2_. The largest lesion size caused by DM118 was under 600 ppm of CO_2_. This was significantly larger than those produced under a normal environment, 400 ppm, and 800 ppm. The D5 isolate caused a significantly larger lesion size when the CO_2_ concentration increased to 800 ppm. In contrast, the lesion size caused by isolate DS103 was significantly smaller under 800 ppm (Figure 1). The lesion size caused by *L. maculans* isolates CDS-13, DM96, and umavr7 had no significant change under the four different CO_2_ concentrations (Figure 1). 

### 2.3. Pathogenicity Evaluation of Compatible and Incompatible Interactions between Leptosphaeria spp. and B. napus

**Figure 2:**
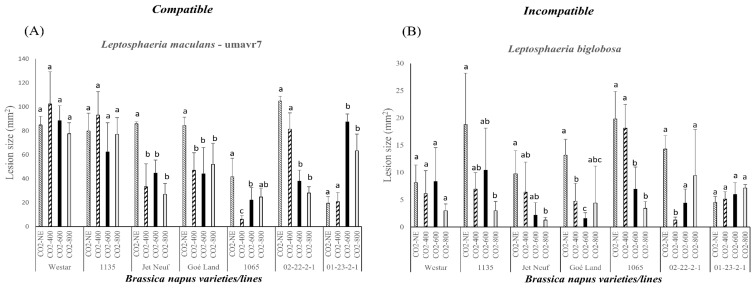
(**A**) Pathogenicity assessment of one virulent *Leptosphaeria maculans* isolate umavr7 carrying none of the corresponding *Avr* genes, showing compatible interaction with all *Brassica napus* varieties and lines under different CO_2_ concentrations (NE, normal environment; CO_2_-400, 400 ppm; CO_2_-600, 600 ppm; CO_2_-800, 800 ppm). (**B**) Pathogenicity assessment of one avirulent or non-aggressive *Leptosphaeria biglobosa* isolate inoculated onto all *B. napus* varieties and lines, showing incompatible reaction under different CO_2_ concentrations. The lesion sizes were quantified at 14 days post-inoculation (dpi) for each treatment using Assess 2.0 (American Phytopathological Society, St. Paul, MN, USA). Statistical analysis was performed using SAS version 9.4 (SAS Institute, Inc., Cary, NC, USA) for analysis of variance (ANOVA). Each bar indicates the mean and standard deviation of the lesion size for a particular isolate in different CO_2_ concentrations. Different letters over the bars represent the significant differences (*p* < 0.05) in the lesion sizes caused by a specific isolate under four different CO_2_ environments.

The two isolates umavr7 and *L. biglobosa*, which have virulence and avirulence, respectively, to all studied *B. napus* varieties and lines, were used to investigate the compatible and incompatible reactions and the effect of CO_2_ concentrations (Table 1 and Figure 2). The lesion sizes were significantly decreased on *B. napus* varieties and lines Jet Neuf (*Rlm4*) and Goé Land (*Rlm9*) inoculated by umavr7 as the CO_2_ concentrations increased (Figure 2A). umavr7 caused significantly smaller lesions on *B. napus* line 02-22-2-1 (*Rlm3*) under 600 and 800 ppm of CO_2_ compared with the normal environment and 400 ppm. In *B. napus* line 1065 (*LepR1*), the lesion size was significantly decreased under 400 ppm of CO_2_. The lesion sizes were all smaller in *B. napus* line 1065 under elevated CO_2_ concentrations compared to those in the normal environment (Figure 2A). However, the increased CO_2_ concentrations enhanced the pathogenicity of umavr7 significantly when inoculated onto *B. napus* line 01-23-2-1 (*Rlm7*) (Figure 2A). There were no significant changes in lesion size when the umavr7 isolate was inoculated onto *B. napus* varieties and lines Westar and 1135 under different CO_2_ concentrations (Figure 1 and Figure 2A).

For the incompatible interaction, the lesion sizes were decreased under the highest CO_2_ concentration (800 ppm) when inoculated onto seven *B. napus* varieties and lines, except 01-23-2-1 (*Rlm7*), compared to those under the normal environment (Figure 2B). For example, the lesion sizes were significantly smaller under 800 ppm CO_2_ when inoculated onto *B. napus* varieties and lines 1135, Jet Neuf, and 1065 (Figure 2B). The smallest lesions caused by the *L. biglobosa* isolate from *B. napus* line Goé Land and 02-22-2-1 were under CO_2_ concentrations of 600 and 400 ppm, respectively (Figure 2B). As in the compatible interaction, the higher CO_2_ concentration enhanced the pathogenicity of the *L. biglobosa* isolate when inoculated onto *B. napus* line 01-23-2-1 (Figure 2A,B). 

### 2.4. Pathogenicity Evaluation of Compatible and Incompatible Interactions on Different Brassica napus Varieties and Lines Caused by Leptosphaeria maculans

**Figure 3:**
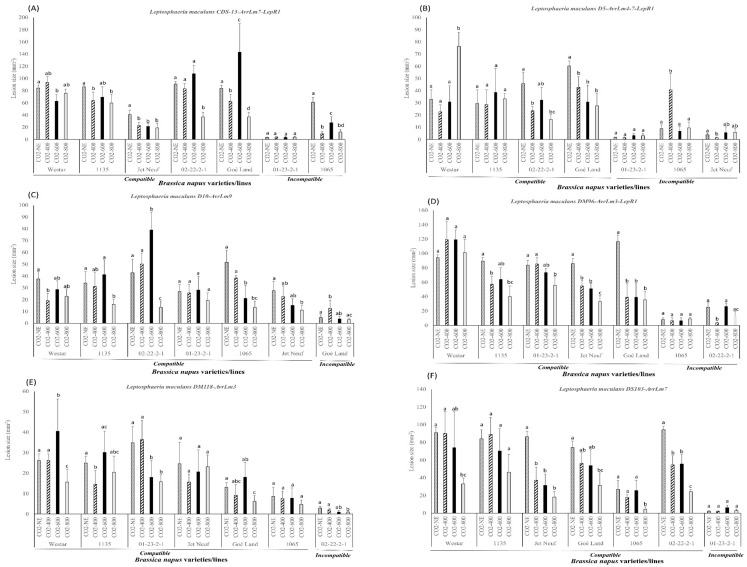
Pathogenicity assessment of virulent *Leptosphaeria maculans* isolates carrying different *Avr* gene profiles and showing compatible and incompatible reactions on corresponding *Brassica napus* varieties and lines under different CO_2_ concentrations (NE, normal environment; CO_2_-400, 400 ppm; CO_2_-600, 600 ppm; CO_2_-800, 800 ppm). The *L. maculans* isolates used in this study were as follows: (**A**) CDS-13; (**B**) D5; (**C**) D10; (**D**) DM96; (**E**) DM118; and (**F**) DS103. The lesion sizes were assessed at 14 days post-inoculation (dpi) for each treatment using Assess 2.0 (American Phytopathological Society, St. Paul, MN, USA). Statistical analysis was performed using SAS version 9.4 (SAS Institute, Inc., Cary, NC, USA) for analysis of variance (ANOVA). Each bar indicates the mean and standard deviation of the lesion size for a particular isolate in different CO_2_ concentrations. Different letters over the bars represent the significant differences (*p* < 0.05) of the lesion sizes caused by a specific isolate under four different CO_2_ environments.

*L. maculans* isolates showed compatible and incompatible interactions with the different *B. napus* varieties and lines since they carried different *Avr* gene profiles corresponding to the resistance genes (Figure 3). For example, *L. maculans* isolate CDS-13 showed compatible reactions on the *B. napus* varieties and lines Westar, 1135, Jet Neuf, 02-22-2-1, and Goé Land; however, it displayed incompatible interactions with 1065 and 01-23-2-1 (Figure 3A). Similarly, *L. maculans* isolates D5, D10, DM96, DM118, and DS103 showed various interactions on different *B. napus* varieties and lines (Figure 3). As shown in Figure 3, most of the lesion sizes caused by *L. maculans* isolates decreased at the extremely high CO_2_ concentration (800 ppm) compared with those under the normal environment. Only isolates D5 and DM96 caused larger lesions on *B. napus* variety Westar at the extremely high CO_2_ concentration (Figure 3B).

For each *L. maculans* isolate, CDS-13 caused a significantly smaller lesion size on *B. napus* varieties and lines 1135, Jet Neuf, 02-22-2-1, and 1065 at the CO_2_ concentration of 800 ppm. On *B. napus* line Goé Land, CDS-13 caused a significantly larger lesion size at 600 ppm of CO_2_. As well, lesions caused by the CDS-13 isolate were larger on *B. napus* line 02-22-2-1 at 600 ppm. The lesion sizes on *B. napus* line Jet Neuf caused by CDS-13 were significantly decreased when CO_2_ concentration increased (Figure 3A). *L. maculans* isolate D5 caused significantly smaller lesions on *B. napus* line Goé Land when the CO_2_ concentrations increased. The lesion sizes on *B. napus* varieties and lines 02-22-2-1 and Jet Neuf caused by D5 were significantly decreased at 400 ppm CO_2_ (Figure 3B). *L. maculans* isolate D10 caused significantly decreased lesion sizes on *B. napus* varieties and lines 1135, 02-22-2-1, 1065, and Jet Neuf under a CO_2_ concentration of 800 ppm. It caused no significant change on the *B. napus* line Goé Land, showing an incompatible reaction under extremely high CO_2_ concentration (800 ppm). The largest lesion size on *B. napus* line 02-22-2-1 caused by D10 was under 600 ppm CO_2_ (Figure 3C). DM96 caused significantly smaller lesion sizes on *B. napus* varieties and lines 1135, 01-23-2-1, Jet Neuf, and Goé Land at 800 ppm of CO_2_. It caused the smallest lesions on 02-22-2-1 at 400 ppm CO_2_ and showed an incompatible reaction with the *AvrLm3* gene (Figure 3D). *L. maculans* isolates DM118 and DS103, carrying *AvrLm3* and *AvrLm7*, only showed incompatible interactions on *B. napus* varieties and lines 02-22-2-1 (*Rlm3*) and 01-23-2-1 (*Rlm7*), respectively (Figure 3E,F). DM118 induced significantly smaller lesions on *B. napus* varieties and lines 01-23-2-1, Goé Land, and 02-22-2-1 at a CO_2_ concentration of 800 ppm, whereas DS103 caused significantly smaller lesions on *B. napus* varieties and lines Jet Neuf, 1065, and 02-22-2-1 (Figure 3E,F). The lesion sizes were decreased at 400 ppm and then increased at 600 ppm when DM118 was inoculated onto *B. napus* varieties and lines 1135, Jet Neuf, and Goé Land (Figure 3E). DS103 caused a steady decline of lesion sizes on most of the *B. napus* varieties and lines when the CO_2_ concentration was elevated (Figure 3F).

## 3. Discussion

Plants usually benefit by increasing the photosynthetic products and/or utilization efficiencies of water and nutrients when growing under rising temperatures and CO_2_ concentrations [35]. However, the effects of elevated CO_2_ concentration on plant–pathogen interactions have undergone limited studies, producing inconsistent results [13,15,16,17,18,36]. In this study, we aimed to investigate how elevated CO_2_ concentration affects the canola–blackleg interaction using different *B. napus* varieties and lines with different *R* genes and *Leptosphaeria* isolates with various *Avr* gene profiles. We found that the CO_2_ concentration has no impact on the inferred phenotypes, which are determined by the qualitative resistance (*R* gene) in canola. However, the extremely high CO_2_ concentration (800 ppm) inhibits the pathogenicity of *Leptosphaeria* spp. on the canola plant with the exception of D5 isolate on the susceptible *B. napus* variety Westar. The variability of the enhanced or inhibited pathogenicity in *B. napus–L. maculans* interactions will provide a starting point for future studies on the physiology and genetics of certain *B. napus* lines in response to fungal disease under different CO_2_ concentrations.

*B. napus* has both qualitative (*R* gene resistance in the seedling) and quantitative (adult plant) resistance to blackleg disease [37]. Currently, most of the canola cultivars grown in Canadian canola fields have single or multiple *R* genes (Canola Council of Canada). Therefore, the main resistance of canola seedlings to blackleg disease is determined by the qualitative resistance (*R* gene(s)) [38,39]. There were slight changes in the disease rating scores of different *B. napus* varieties and lines in responding to various *Leptosphaeria* spp. isolates under different elevated CO_2_ concentrations, and these did not shift the inferred phenotypes of the interactions in this study. This result supports the conclusion that the *R* gene-mediated resistance is the major factor for canola seedlings in conferring the environmental element variations (i.e., elevated CO_2_ concentration). This is similar to a previous review that indicated that the abiotic stress tolerance may or may not correlate with the plant’s resistance to disease [40]. There was no change of inferred phenotypes of the susceptible *B. napus* variety Westar conferred by different *Leptosphaeria* isolates under different CO_2_ concentrations. The interactions between canola varieties and lines carrying different *R* genes and *Leptosphaeria* isolates had slight changes, that is, from susceptible to intermediate resistance/susceptible or from resistance to intermediate resistance, indicating that physiological stresses and variations occurred in the host plant or that the fungal pathogen may affect the disease severity aside from the major *R* gene mediation.

Global climate warming caused by the elevated CO_2_ concentration is well documented and widely accepted [2]. Although the elevated CO_2_ concentration has no impact on the major *R* gene-determined resistance of canola seedlings, it should have profound effects on plant and fungi growth, development and reproduction. Therefore, the degree of disease severity quantified by lesion size had significant variations under different CO_2_ concentrations. The main finding of this study was that the lesion sizes were significantly smaller in most of the *B. napus* varieties inoculated with different *L. maculans* isolates at extremely high CO_2_ concentration (800 ppm). The disease incidence or severity was decreased under elevated CO_2_ concentrations in previous studies [13,41,42,43,44]. For example, resistance was induced at 700 ppm and associated with the canopy size in the Fitzroy–*Colletotrichum gloeosporioides* pathosystem [41]. McElrone et al. (2005) found that elevated CO_2_ significantly reduced the disease incidence and severity of a red maple (*Acer rubrum*) fungal pathogen (*Phyllosticta minima*) through the changes in leaf chemistry and host physiology, that is, stomatal conductance reduction and smaller openings for fungi germ tubes or reduced nutritive quality [13]. Conversely, the elevated CO_2_ concentration increased the disease incidence and severity of rice blast (*Magnaporthe oryzae*) and sheath blight (*Rhizoctonia solani*) in rice (*Oryza sativa*), probably because a higher number of tillers was observed, which may increase the chance for sclerotia to adhere to the leaf sheath [45]. The leaf spot disease incidence and severity in redbud (*Cercis canadensis*) and sweetgum trees (*Liquidambar styraciflua*) were increased under elevated CO_2_ concentration, since the enhanced photosynthetic efficiency in the remaining leaf tissues minimized or mitigated any increases of the disease symptoms [16]. An interesting study conducted by Eastburn et al. (2010) found that increased CO_2_ concentration significantly reduced and increased the disease severity of downy mildew and brown spot disease in the soybean, respectively [42]. They also found that CO_2_ or O_3_ treatment had no effects on the incidence of sudden death syndrome (SDS) of the soybean [42]. All these findings indicated that the effects of elevated CO_2_ concentration on the disease incidence and severity depended on the plant and pathogen species. The effects of elevated CO_2_ concentration on physiological processes, leaf chemistry, the stomatal opening system, and even on *L. maculans* growth rate, sporulation and aggressiveness, probably caused the decreased disease severity of canola seedlings inoculated by different *L. maculans* isolates. Further studies are needed to investigate causes of the inhibition of *Leptosphaeria* isolates’ pathogenicity on the canola plant under elevated CO_2_ concentration and at the molecular level.

There were different lesion size variation patterns for the canola plants inoculated with different *L. maculans* isolates carrying different *Avr* gene profiles under CO_2_ concentrations of 400 and 600 ppm. For example, compatible interactions (i.e., CDS-13 on *B. napus* varieties and lines 02-22-1 and Goé Land; D5 on 1135; D10 on *B. napus* varieties and lines 1135, 02-22-2-1, and 01-23-2-1; DM118 on *B. napus* varieties and lines Westar, 1135, and Goé Land) showed largest lesion sizes at an elevated CO_2_ concentration of 600 ppm. Several studies reported that elevated CO_2_ concentration stimulated fungal growth, aggressiveness, sporulation, and fecundity [13,46,47,48]. The increased disease expression at elevated CO_2_ concentration may induce the larger lesions in certain plant species, varieties, or lines. This may be the reason that *L. maculans* isolates displayed increased pathogenicity on *B. napus* varieties and lines specifically at elevated CO_2_ concentration. However, several compatible interactions showed the highest lesion size at a CO_2_ concentration of 400 ppm or a normal environment. For incompatible interactions, the disease expression also increased at different CO_2_ concentrations (normal environment, 400, or 600 ppm), and the disease severity mostly was inhibited at extremely high CO_2_ concentration (800 ppm) (Figure 3). Therefore, the increased pathogenicity of *Leptosphaeria* spp. observed here did not produce a consistent expression pattern under certain CO_2_ concentrations, indicating that the disease development could result from the characteristics of *Leptosphaeria* or other changes in the host plant. It is well known that *L. maculans* isolates usually carry different *Avr* gene profiles, thus they show different pathogenicity and interactions with canola plants. *L. maculans* isolates carrying more *Avr* genes may show less virulence on the *B. napus* varieties and lines since they only cause disease symptoms in the varieties and lines without the corresponding resistance genes. Therefore, the effects of elevated CO_2_ concentration on the effector or elicitor gene in *Leptosphaeria* may differ and induce different disease severity in canola plants. On the other hand, differences in the host (canola) plant traits (i.e., genetic background, resistance genes, plant morphology, and leaf and cotyledon structure) probably initiate various defense mechanisms under high CO_2_ concentration stresses. Long-term studies including fungal genetics and biology, plant physiology, and genetics, etc., are needed to disclose the mechanisms of *B. napus* and *Leptosphaeria* spp. interactions under elevated CO_2_ concentration.

Global warming at present and in the future could reduce the disease severity of the canola–blackleg pathosystem through elevated CO_2_ at an extremely high level of 800 ppm. However, in this study, the pathogenicity of *Leptosphaeria* spp. seems increased in a couple of interactions with different *B. napus* varieties and lines at 400 ppm or 600 ppm CO_2_. We cannot make specific predictions on the change patterns from field trials since the CO_2_ concentration can be easily controlled only in a growth chamber. The *B. napus* varieties and lines used in this study are differential lines used for the characterization of *Avr* gene profiling in *L. maculans* isolates. We were trying to understand the effects of elevated CO_2_ concentration on different resistant gene (*R*) in response to various *L. maculans* isolates harboring different *Avr* genes. Since the most commercial canola varieties in fields in Canada have the *Rlm3* gene [38], it is not practical to use cultivars with one *R* gene to represent the effects of elevated CO_2_ concentration. However, the results obtained from this study provide a clue of how elevated CO_2_ concentration affects the *R*–*Avr* interactions of the *B. napus–Leptosphaeria maculans* pathosystem in both compatible and incompatible reactions. The numbers of *B. napus* varieties and lines and *L. maculans* isolates employed in this study will also trigger a discussion of how the different canola plant varieties show different resistance mechanisms in response to the different *Leptosphaeria* spp. infections under elevated CO_2_ concentration.

## 4. Materials and Methods

### 4.1. Brassica napus Varieties and Lines and Leptosphaeria maculans Isolates

*B. napus* varieties and lines carrying known *R* genes were used to test the compatible and incompatible interactions with different isolates (Table 1). For example, *B. napus* line 02-22-2-1, having the *Rlm3* gene, showed compatible interaction with the isolate without the *AvrLm3* gene, while showing incompatible reaction with the isolate carrying the *AvrLm3* gene. Accordingly, several other *B. napus* varieties and lines (i.e., Jet Neuf, 01-23-2-1, Goé Land, 1065, and 1135 harboring *Rlm4*, *Rlm7*, *Rlm9*, *LepR1*, and *LepR2*, respectively) were included in this study (Table 1). These *B. napus* varieties and lines from different countries (Canada, France, and others) were extensively used for the characterization of *Avr* gene profiles in *L. maculans* isolates [38]. A susceptible *B. napus* variety Westar, which is a spring canola variety that has none of the resistant genes to blackleg disease, was also included as a control (Table 1).

The previously characterized International Blackleg of Crucifers Network (IBCN) *L. maculans* isolates D5 and D10 having *AvrLm1-2-4-7-S-LepR1-LepR2* and *AvrLm5-6-9-LepR1*, respectively, were used in this study [20,38]. Five isolates from canola fields of Western Canada, namely, CDS-13, DM96, DM118, DS103, and umavr7, harboring *AvrLm5-6-7-LepR1*, *AvrLm2-3-6-S*, *AvrLm3-6-S*, *AvrLm5-6-7*, and *AvrLm5-6*, respectively, were included as well. A *L. biglobosa* isolate, which is the non-aggressive or avirulent form of *Leptosphaeria* spp. to the canola plant, was used as a control in this study.

### 4.2. Plant Cotyledon Inoculation and CO_2_ Treatment

The *Leptosphaeria* spp. isolate inocula were harvested by flooding eleven-day-old cultures from a single pycnidiospore using distilled water. The single pycnidiospore was picked from culture of a stocked paper disc of each isolate [38,39]. The final concentration of inoculum of each isolate was adjusted to 2 × 10^7^ spores/mL for the later cotyledon inoculation test. The seven *B. napus* varieties and lines with or without different *R* genes were seeded into 96-well flats (53 × 27× 7 cm as length, width and height) filled with commercialized soil (Pro-Mix BX, Premier Tech, Rivière-du-Loup, QC, Canada) and placed in a growth chamber at 16 °C (night) and 21 °C (day) with a 16 h photoperiod/day. The seven-day-old seedlings were used for inoculation and grown in different CO_2_ concentrations. The cotyledons were punctured and inoculated with a prepared 10 µL inoculum droplet at each of two wound sites per cotyledon [38]. Therefore, at least 24 wound sites from 6 plants were inoculated and rated at 14 days post-inoculation.

Four flats of each of the *B. napus* varieties and lines were inoculated with different *Leptosphaeria* isolates. After one or two hours drying, one flat of one *B. napus* variety or line was moved to the growth chamber with normal growth conditions after inoculation of *Leptosphaeria* isolates. To investigate the elevated and extremely high CO_2_ concentration effects on disease severity, the other three flats with each of the *B. napus* varieties and lines were placed into controlled-environment growth chambers with different CO_2_ concentrations of 400, 600, and 800 ppm, respectively, with a 16 °C/21 °C night/day and a 16 h photoperiod/day (Conviron, Winnipeg, MB, Canada). The CO_2_ concentration of each growth chamber was monitored by CO_2_/Temp./RH Data Logger (Professional Instruments, Huizhou, Guangdong, China). All the experiments were repeated once.

### 4.3. Disease Rating and Lesion Size Measurement

Based on the lesions, chlorosis, necrosis and signs of pycnidia, a rating scale from 0 to 9 was used to evaluate the disease severity at 14 days post-inoculation [38,39]. An average score was calculated from 24 inoculation sites of six plants (four wound sites per plant). Therefore, an average rating score ≤ 4.5 was considered as a resistant (R) reaction, 4.6 to 6.0 as an intermediate resistance (IR) reaction and 6.1 to 9.0 as a susceptible (S) reaction [38,49]. The lesion sizes of the infected cotyledons, under different CO_2_ concentrations for each variety or line with different isolates, were quantified at 14 dpi using Assess 2.0 image software (American Phytopathological Society, St. Paul, MN, USA).

### 4.4. Statistical Methodology

Statistical analysis of the lesion sizes caused by the different isolates under different CO_2_ concentrations, and the F measurement of distance between two repeats, were conducted using SAS version 9.4 (SAS Institute, Inc., Cary, NC, USA). The data were subjected to ANOVA and the mean lesion sizes were compared by Tukey’s HSD studentized range test at *p* < 0.05.

## Figures and Tables

**Table 1:** Mean rating scores and their inferred disease resistance for seedlings of *Brassica napus* varieties and lines in response to different *Leptosphaeria* spp. isolates under different CO_2_ concentrations.

		Disease Rating							
*B. napus* Varieties and Lines	CO_2_	CDS-13 ^d^	D5 ^d^	D10 ^d^	DM96 ^d^	DM118 ^d^	DS103 ^d^	umavr7 ^d^	LB ^e^
Westar/No *R* gene ^a^	NE ^b^	7.25 (S) ^c^	7.33 (S)	7.50 (S)	8.00 (S)	7.00 (S)	9.00 (S)	8.33 (S)	3.50 (R)
	400	8.33 (S)	7.50 (S)	9.00 (S)	7.25 (S)	8.33 (S)	7.55 (S)	9.00 (S)	2.67 (R)
	600	8.00 (S)	8.00 (S)	8.33 (S)	7.00 (S)	9.00 (S)	8.00 (S)	7.50 (S)	3.33 (R)
	800	7.50 (S)	8.33 (S)	6.25 (S)	6.83 (S)	6.15 (S)	7.00 (S)	6.83 (S)	2.33 (R)
1065/*LepR1* ^a^	NE	8.00 (S)	4.25 (R)	7.87 (S)	3.33 (R)	5.85 (IR)	7.50 (S)	8.33 (S)	4.87 (IR)
	400	8.33 (S)	6.00 (S)	7.50 (S)	3.00 (R)	5.25 (IR)	6.83 (S)	5.25 (IR)	4.50 (R)
	600	7.50 (S)	4.60 (R)	7.50 (S)	2.87 (R)	6.00 (S)	7.15 (S)	6.67 (S)	3.87 (R)
	800	7.50 (S)	5.00 (IR)	6.67 (S)	2.87 (R)	5.00 (IR)	5.25 (IR)	8.00 (S)	3.00 (R)
1135/*LepR2* ^a^	NE	8.33 (S)	6.67 (S)	7.00 (S)	8.13 (S)	7.00 (S)	8.33 (S)	7.50 (S)	5.25 (IR)
	400	7.50 (S)	6.50 (S)	6.50 (S)	7.50 (S)	6.83 (S)	9.00 (S)	8.00 (S)	4.50 (R)
	600	6.67 (S)	7.00 (S)	6.67 (S)	7.87 (S)	8.33 (S)	8.00 (S)	6.87 (S)	5.00 (IR)
	800	7.00 (S)	6.50 (S)	5.65 (IR)	6.87 (S)	7.00 (S)	6.25 (S)	7.33 (S)	3.87 (R)
02-22-2-1/*Rlm3* ^a^	NE	8.33 (S)	7.50 (S)	6.67 (S)	4.00 (R)	2.67 (R)	8.33 (S)	8.33 (S)	5.00 (IR)
	400	8.00 (S)	6.00 (S)	7.33 (S)	2.33 (R)	1.67 (R)	7.86 (S)	7.50 (S)	4.50 (R)
	600	8.00 (S)	6.67 (S)	9.00 (S)	3.67 (R)	1.33 (R)	7.50 (S)	5.75 (IR)	3.33 (R)
	800	6.83 (S)	6.00 (S)	5.33 (IR)	3.00 (R)	1.33 (R)	6.83 (S)	5.00 (IR)	3.00 (R)
01-23-2-1/*Rlm7* ^a^	NE	1.33 (R)	2.13 (R)	6.50 (S)	7.50 (S)	7.25 (S)	1.50 (R)	6.25 (S)	2.67 (R)
	400	2.67 (R)	2.50 (R)	6.67 (S)	7.13 (S)	8.00 (S)	2.33 (R)	7.00 (S)	3.00 (R)
	600	2.67 (R)	3.00 (R)	7.00 (S)	7.00 (S)	6.87 (S)	3.00 (R)	9.00 (S)	3.00 (R)
	800	2.33 (R)	1.67 (R)	6.33 (S)	6.87 (S)	6.25 (S)	2.33 (R)	8.15 (S)	3.00 (R)
Jet Neuf/*Rlm4* ^a^	NE	7.50 (S)	1.67 (R)	8.15 (S)	8.15 (S)	8.25 (S)	8.33 (S)	8.33 (S)	4.00 (R)
	400	6.87 (S)	1.33 (R)	8.00 (S)	7.50 (S)	6.87 (S)	8.00 (S)	6.67 (S)	3.50 (R)
	600	6.00 (S)	2.50 (R)	7.50 (S)	7.50 (S)	7.50 (S)	7.86 (S)	7.15 (S)	3.00 (R)
	800	6.50 (S)	2.00 (R)	6.00 (S)	7.00 (S)	7.86 (S)	6.83 (S)	7.50 (S)	2.33 (R)
Goé Land/*Rlm9* ^a^	NE	7.50 (S)	8.15 (S)	2.50 (R)	8.33 (S)	8.33 (S)	7.86 (S)	8.15 (S)	5.00 (IR)
	400	7.33 (S)	7.50 (S)	3.50 (R)	6.87 (S)	7.00 (S)	7.00 (S)	7.33 (S)	4.13 (R)
	600	8.33 (S)	7.00 (S)	2.13 (R)	6.50 (S)	8.33 (S)	6.83 (S)	7.00 (S)	3.50 (R)
	800	7.00 (S)	6.67 (S)	1.87 (R)	6.00 (S)	6.67 (S)	6.33 (S)	6.83 (S)	3.00 (R)

^a^*Brassica napus* varieties and lines carrying different resistance genes used in this study. ^b^ The seedlings were grown in four different controlled environments, namely, NE means normal environment and 400, 600, 800 ppm indicate the CO_2_ concentration of the growth chambers, respectively. ^c^ R, IR, S = *Brassica napus* varieties and lines that displayed by averaged rating scores as resistant (≤ 4.5), intermediate resistance (4.6 to 6.0) and susceptible (6.1 to 9.0), respectively, to different *Leptosphaeria* isolates under different CO_2_ concentrations. ^d^ indicates *L. maculans* isolates. ^e^ LB indicates *L. biglobosa* isolate.
